# Prediction and Identification of Power Performance Using Polygenic Models of Three Single-Nucleotide Polymorphisms in Chinese Elite Athletes

**DOI:** 10.3389/fgene.2021.726552

**Published:** 2021-10-08

**Authors:** Ruoyu Yang, Feng Jin, Liyan Wang, Xunzhang Shen, Qi Guo, Haihan Song, Jingyun Hu, Qiang Zhao, Jian Wan, Ming Cai

**Affiliations:** ^1^ College of Rehabilitation Sciences, Shanghai University of Medicine and Health Sciences, Shanghai, China; ^2^ Department of Talent Identification and Development, Shanghai Research Institute of Sports Science (Shanghai Anti-Doping Center), Shanghai, China; ^3^ Central Lab, Shanghai Pudong New Area People’s Hospital, Shanghai, China; ^4^ National Center for Gene Research, CAS Center for Excellence in Molecular Plant Sciences, Chinese Academy of Sciences, Shanghai, China; ^5^ Department of Emergency and Critical Care Medicine, Shanghai Pudong New Area People’s Hospital, Shanghai, China; ^6^ Shanghai University of Medicine and Health Sciences Affiliated Zhoupu Hospital, Shanghai, China

**Keywords:** gene polymorphism, talent identification, athletic status, physical performance, nomogram

## Abstract

**Objective:** The manuscript aims to explore the relationship between power performance and SNPs of Chinese elite athletes and to create polygenic models.

**Methods:** One hundred three Chinese elite athletes were divided into the power group (*n* = 60) and endurance group (*n* = 43) by their sports event. Best standing long jump (SLJ) and standing vertical jump (SVJ) were collected. Twenty SNPs were genotyped by SNaPshot. Genotype distribution and allele frequency were compared between groups. Additional genotype data of 125 Chinese elite athletes were used to verify the screened SNPs. Predictive and identifying models were established by multivariate logistic regression analysis.

**Results:** ACTN3 (rs1815739), ADRB3 (rs4994), CNTFR (rs2070802), and PPARGC1A (rs8192678) were significantly different in genotype distribution or allele frequency between groups (*p* < 0.05). The predictive model consisted of ACTN3 (rs1815739), ADRB3 (rs4994), and PPARGC1A (rs8192678), the area under curve (AUC) of which was 0.736. The identifying model consisted of body mass index (BMI), standing vertical jump (SVJ), ACTN3, ADRB3, and PPARGC1A, the area under curve (AUC) of which was 0.854. Based on the two models, nomograms were created to visualize the results.

**Conclusion:** Two models can be used for talent identification in Chinese athletes, among which the predictive model can be used in adolescent athletes to predict development potential of power performance and the identifying one can be used in elite athletes to evaluate power athletic status. These can be applied quickly and visually by using nomograms. When the score is more than the 130 or 148 cutoff, it suggests that the athlete has a good development potential or a high level for power performance.

## Introduction

The physical performance and athletic capacity of elite athletes, such as endurance, power, speed, flexibility, and sensitivity, are influenced by many factors, among which genetic factors are important (Puthucheary et al., 2011; Ahmetov et al., 2016; Peplonska et al., 2017). It is a major task and research direction to use genetic factors to predict the development potential of physical performance and athletic capacity in adolescent athletes and to evaluate and identify the level of elite athletes (Breitbach et al., 2014; Webborn et al., 2015; Pickering et al., 2019).

In physical performance and athletic capacity, power performance has a relatively high heritability (Maciejewska-Skrendo et al., 2019), which is particularly important for the power-orient sports and critical in talent identification (Yang et al., 2017). It has been reported that the estimated heritability of muscle strength and mass varies from approximately 30–80% with large differences of muscle groups, contraction velocities, and muscle lengths (Peeters et al., 2009). Therefore, these genetic variants may contribute to elite athletic performance such as power. Using these genetic variants, it is possible to identify future elite athletes. Single nucleotide polymorphism (SNP) is a form of genetic variants, which is often used to study and investigate the genetic factors of physical performance (Sarzynski et al., 2016). SNP mainly refers to the DNA sequence polymorphism caused by single nucleotide variation at the genomic level. It is one of the most common human heritable variations, accounting for more than 90% of all known polymorphisms. SNPs exist widely in the human genome, with an average of one in every 300 base pairs. It is estimated that the total number of SNPs can reach 3 million or more. SNP can result from the transition or transversion of a single base, or from the insertion or deletion of a base (Brookes, 1999). For example, a SNP of R577X (rs1815739) in the ACTN3 gene modifies the attainment of elite power-oriented athletic performance status (Yang et al., 2017; Tharabenjasin et al., 2019). In this SNP, common C-to-T base substitution results in the transformation of an arginine base (R) to a premature stop codon (X). X allele homozygotes are deficient in the protein encoded by ACTN3, *α*-actinin-3, which is expressed exclusively in fast-twitch muscle fibers. As a result, XX genotypes tend to have lower proportions of fast-twitch muscle fibers (Vincent et al., 2007), and decreased power performance, because fast-twitch muscle fiber is an important component of power-oriented performance (Pickering et al., 2019). In fact, physical performance may be influenced by hundreds of related SNPs (Ben-Zaken et al., 2015; Grealy et al., 2015). Previous polygenic studies did not differentiate the role of SNPs in prediction and identification; it is therefore hard to be applied in talent identification (Ruiz et al., 2010; Buxens et al., 2011).

Based on the above, we recruited Chinese elite athletes to explore the relationship between polygenic profiles and elite power performance. We then attempted to establish the predictive and identifying models, based on which nomograms were also created for talent identification in adolescent and elite athletes.

## Material and Methods

### Ethics Approval

This study was conducted on the basis of the Declaration of Helsinki and approved by the ethics committee of the School of Life Sciences Fudan University (Shanghai, China). Before the study, written informed consent was obtained from all participants.

### Participants

A total of 103 elite Chinese athletes of Han origin (age = 24.3 ± 3.2 years, male = 24.5 ± 3.5 years, female = 24.1 ± 2.8 years; height = 179.3 ± 9.3 cm, male = 183.3 ± 7.6 cm, female = 172.6 ± 7.8 cm; weight = 75.8 ± 15.4 kg, male = 81.2 ± 15.2 kg, female = 66.8 ± 11.2 kg; body mass index (BMI) = 23.4 ± 3.5 kg/m^2^, male = 24.1 ± 3.7 kg/m^2^, female = 22.4 ± 3.0 kg/m^2^) were recruited and categorized into the power group (*n* = 60) and endurance group (*n* = 43) as determined by the distance, duration, and energy requirements of their sports event. Athletes in the power group included 100-m and 200-m sprinters (*n* = 27), jumpers (*n* = 14), shot putters (*n* = 7), discus throwers (*n* = 4), javelin throwers (*n* = 2), weightlifters (*n* = 2), and 500-m track sprint cyclists (*n* = 4), while the endurance group included 5,000-m runners (*n* = 19), 10,000-m runners (*n* = 20), and marathon runners (*n* = 4) whose events demand predominantly aerobic energy production. These elite athletes were all national-master and international-master athletes with certification by national specialized departments of China.

An additional 125 elite athletes were selected to repeat significant SNPs and validate models crossly. These athletes were also from the high-level sports team in Shanghai. Seventy-three power athletes (age = 21.2 ± 1.9 years, male = 21.3 ± 1.8 years, female = 21.1 ± 2.1 years; body mass index (BMI) = 20.9 ± 1.5 kg/m^2^, male = 21.3 ± 1.3 kg/m^2^, female = 20.1 ± 1.5 kg/m^2^) were from sprinting, throwing and jumping events and 52 endurance athletes (age = 20.1 ± 1.5 years, male = 20.2 ± 1.7 years, female = 20.0 ± 1.4 years; body mass index (BMI) = 19.7 ± 1.2 kg/m^2^, male = 20.1 ± 1.3 kg/m^2^, female = 19.2 ± 1.0 kg/m^2^) were from middle and long distance running, who were also all Chinese Han origin.

### Standing Long Jump and Standing Vertical Jump Data Collection

The athletes’ best standing long jump (SLJ) and standing vertical jump (SVJ) results were collected retrospectively in the Oriental Land Training Base (Shanghai, China). All tests were conducted with a standardized protocol employed at the training base. Specifically, the SLJ was assessed on the test pad special for standing long jump. Subjects started the test with their toes behind the test line; the distance from the rearmost heel strike to the starting line was used for measurement. Three trials were allowed for each subject to achieve their maximal jump performance. The SVJ results were recorded by a standardized electronic vertical jump tester (Jianmin, Beijing, China). Subjects stood on the test board with the location marked and attempted to reach the maximum height vertically, with a landing point within 10 cm from the starting point. The examiner recorded the height displayed from the electronic screen, and the highest two of the three jumps were used. Before all testing, subjects completed a supervised warm-up of running and dynamic stretching, and more specific, submaximal jump warm-up protocol (Ahmetov et al., 2016).

### Selection of Genetic Polymorphisms

Some reviews revealed that more than 40 SNPs (Ahmetov and Fedotovskaya, 2015) and 69 SNPs (Naureen et al., 2020) are associated with power performance. These reports of SNPs are generally in European ancestry populations and fewer in the Chinese Han population. On the basis of these SNPs, we retained the ones with frequency distribution differences reported in the general population or disease population of Chinese Han nationality and removed the ones without frequency distribution differences or unreported in Chinese Han population as candidate gene markers in this study. Finally, 20 gene polymorphisms (within 17 different genes) were considered to be associated with physical performance and exercise-related phenotypes because the HIF1A (rs28708675) genotype didn’t have a frequency distribution in the power or endurance athletes (all subjects were AA genotype). Sixteen genes and 19 polymorphisms were analyzed and listed in Table 1.

**TABLE 1:** Genotypes and allele frequencies in Chinese elite power and endurance athletes.

Gene symbol	Polymorphism	Genotypes (*n*)		Test for genotypes			Allele (*n*,%)		Test for allele		
		Power (*n* = 60)	Endurance (*n* = 43)	*χ* ^2^	p	adj. p	Power	Endurance	*χ* ^2^	p	adj. p
ABCC8	A/G, rs5210	AA (13) AG (30) GG (17)	AA (10) AG (20) GG (13)	0.122	0.941	0.995	A (56,46.7) G (64,53.3)	A (40,46.5) G (46,53.5)	<0.001	0.982	0.982
ACE	Intron 16 ins/del, rs4646994287	II (28) ID (24) DD (8)	II (15) ID (21) DD (7)	1.430	0.489	0.774	I (80,66.7) D (40,33.3)	I (51,59.3) D (35,40.7)	1.173	0.279	0.982
ACTN3	Arg (R)577Ter (X), rs1815739	RR (28) RX (29) XX (3)	RR (15) RX (16) XX (12)	10.568	**0.005**	0.095	R (85,70.8) X (35,29.2)	R (46,53.5) X (40,46.5)	6.510	**0.011**	0.105
ADRA2A	A/G, rs553668	AA (16) AG (30) GG (14)	AA (8) AG (26) GG (9)	1.268	0.530	0.775	A (62,51.7) G (58,48.3)	A (42,48.8) G (44,51.2)	0.160	0.689	0.982
ADRB3	Trp64Arg (T/C), rs4994	TT (51) TC (9) CC (0)	TT (26) TC (16) CC (1)	8.503	**0.014**	0.133	T (111,92.5) C (9,7.5)	T (68,79.1) C (18,20.9)	7.934	**0.005**	0.095
BDKRB2	-58T/C exon 1 I/D, rs72348790	II (0) ID (2) DD (58)	II (0) ID (1) DD (42)	0.090	0.764	0.995	I (2,1.7) D (118,98.3)	I (1,1.2) D (85,98.8)	0.089	0.766	0.982
CKMM	A/G, rs8111989	AA (40) AG (20) GG (0)	AA (30) AG (12) GG (1)	1.668	0.434	0.774	A (100,83.3) G (20,16.7)	A (72,83.7) G (14,16.3)	0.005	0.941	0.982
CNTFR	T/A, rs2070802	TT (55) TA (5) AA (0)	TT (41) TA (0) AA (2)	6.410	**0.041**	0.260	T (115,95.8) A (5,4.2)	T (82,95.3) A (4,4.7)	0.028	0.867	0.982
	C/T, rs3808871	CC (34) CT (23) TT (3)	CC (31) CT (11) TT (1)	2.640	0.267	0.774	C (91,75.8) T (29,24.2)	C (73,84.9) T (13,15.1)	2.528	0.112	0.532
FABP2	G/A, rs1799883	GG (25) GA (31) AA (4)	GG (18) GA (23) AA (2)	0.191	0.909	0.995	G (81,67.5) A (39,32.5)	G (59,68.6) A (27,31.4)	0.028	0.867	0.982
GNB3	C825T, rs5443	CC (15) CT (32) TT (13)	CC (11) CT (23) TT (9)	0.010	0.995	0.995	C (62,51.7) T (58,48.3)	C (45,52.3) T (41,47.7)	0.009	0.926	0.982
HFE	His63Asp (C/G), rs1799945	CC (57) CG (3) GG (0)	CC (39) CG (4) GG (0)	0.732	0.392	0.774	C (117,97.5) G (3,2.5)	C (82,95.3) G (4,4.7)	0.706	0.401	0.982
KDR	Gln472His (T/A), rs1870377	TT (11) TA (37) AA (12)	TT (12) TA (20) AA (11)	2.417	0.299	0.774	T (59,49.2) A (61,50.8)	T (44,51.2) A (42,48.8)	0.080	0.778	0.982
NRF2	A/G, rs7181866	AA (36) AG (22) GG (2)	AA (26) AG (17) GG (0)	1.489	0.475	0.774	A (94,78.3) G (26,21.7)	A (69,80.2) G (17,19.8)	0.109	0.741	0.982
PPARGC1A	Gly482Ser (A/G), rs8192678	AA (16) AG (30) GG (14)	AA (4) AG (23) GG (16)	5.605	0.061	0.290	A (62,51.7) G (58,48.3)	A (31,36.0) G (55,64.0)	4.936	**0.026**	0.165
SLC9A9	C/T, rs2800	CC (19) CT (33) TT (8)	CC (13) CT (24) TT (6)	0.027	0.987	0.995	C (71,59.2) T (49,40.8)	C (50,58.1) T (36,41.9)	0.022	0.883	0.982
	C/T, rs2801	CC (19) CT (33) TT (8)	CC (13) CT (24) TT (6)	0.027	0.987	0.995	C (71,59.2) T (49,40.8)	C (50,58.1) T (36,41.9)	0.022	0.883	0.982
VDR Apal	C/A, rs7975232	CC (32) CA (17) AA (11)	CC (20) CA (17) AA (6)	1.474	0.479	0.774	C (81,67.5) A (39,32.5)	C (57,66.3) A (29,33.7)	0.034	0.854	0.982
VDR BsmI	G/A, rs1544410	GG (58) GA (2) AA (0)	GG (40) GA (3) AA (0)	0.720	0.396	0.774	G (118,98.3) A (2,1.7)	G (83,96.5) A (3,3.5)	0.702	0.402	0.982

Adj. p: *p* value after FDR (false discovery rate) correction by Benjamini-Hochberg procedure.

Bold values: *p* < 0.05.

### Genotyping

Saliva samples were collected *via* the bio-sample collection kit (Applied Halo Biomat Tech, Suzhou, China) and stored in a −20°C freezer until further use. DNA was extracted from saliva by QIAGEN silica gel adsorption kit (Qiagen Inc., Valencia, CA, United States). Genotyping was conducted by multiplex SNaPshot technology using an ABI fluorescence-based assay allelic discrimination method (Applied Biosystems, Foster City, CA, United States) as described previously (Ahmetov et al., 2016). Briefly, multiplexed PCR and multiplexed single-base extension reactions were first conducted, followed by capillary electrophoresis. PCR multiplexes were conducted in a 10-µl reaction, including 5 µl of SNaPshot Multiplex Kit solution (Applied Biosystems), 2 µl of purified PCR product, 1 µl of the extension primer, and 2 µl of high-purity water. The products of the SNaPshot were processed with ABI3730XL (Applied Biosystems) and data from 20 gene loci variants were analyzed with Gene Mapper Analysis Software, version 4.1 (Applied Biosystems). Genotypes were assessed independently by two investigators blinded to the study. As a quality control measure, 10% of randomly selected DNA samples were analyzed at least twice, and the results were 100% concordant.

### Data Analysis

Chi-Squared ( ${\text{χ}}^{2}$ ) tests were used to test for Hardy–Weinberg equilibrium. ${\text{χ}}^{2}$ Tests were also employed to compare the genotype distribution and allele frequencies of SNPs between the groups in training and validation cohorts, and *p* values were adjusted using the Benjamini and Hochberg multiple comparison test. Odds ratios (OR) were calculated to determine the dominant genotype in SNPs screened out. Univariate logistic regression analysis was performed to calculate the significance and strength of the association between each factor including SNPs and athletic status, and multivariate logistic regression analysis was performed to screen models subsequently. Multivariate logistic regression analysis was also conducted to determine the factors independently associated with athletic status. The predictive model only contained SNPs and the identifying model contained SNPs and other phenotypes, which both crossly validated. The results of multivariate analysis were reported as odds ratios and 95% confidence intervals, and *p* < 0.05 was considered to indicate statistical significance in the multivariate analysis. After these analyses, nomograms were built according to the results for predictive and identifying polygenic models of power performance. Data were analyzed using SPSS 26.0 for Windows and R 4.1.0 version. *p* < 0.05 was considered statistically significant.

## Results

### Determination of Single-Nucleotide Polymorphisms Related to Power Performance

All the gene variants were in Hardy–Weinberg equilibrium (*p* > 0.05). There were significant differences in genotype distribution or allele frequency of four SNPs: ACTN3 (rs1815739), ADRB3 (rs4994), CNTFR (rs2070802), and PPARGC1A (rs8192678) (*p* < 0.05); however, there was no significant difference in all SNPs with the correction of multiple comparison test based on the method of Benjamini and Hochberg (Benjamini and Hochberg, 1995) (adj. *p* > 0.05) (Table 1). To exclude false positives, the screened SNPs would be repeatedly verified by using additional data.

### Validating Candidate Single-Nucleotide Polymorphisms

**FIGURE 1:**
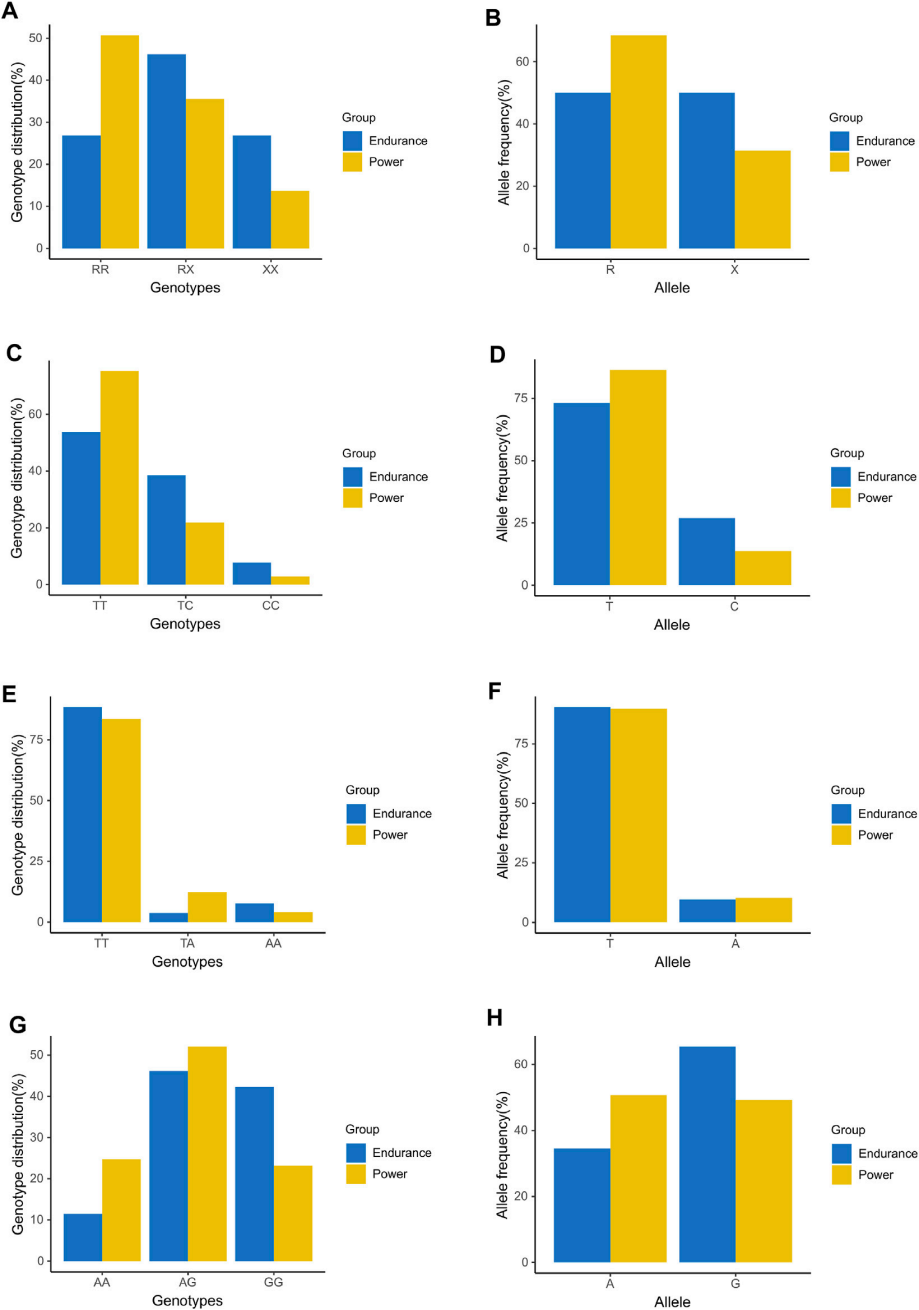
Comparison analysis of genotype distribution and allele frequencies between additional power and endurance athletes in candidate SNPs for validation. **(A)** Genotype distribution of ACTN3 (rs1815739). **(B)** Allele frequency of ACTN3 (rs1815739). **(C)** Genotype distribution of ADRB3 (rs4994). **(D)** Allele frequency of ADRB3 (rs4994). **(E)** Genotype distribution of CNTFR (rs2070802). **(F)** Allele frequency of CNTFR (rs2070802). **(G)** Genotype distribution of PPARGC1A (rs8192678). **(H)** Allele frequency of PPARGC1A (rs8192678).

As external validation data set, the genotype data of 125 elite athletes were used to verify the candidate SNPs: ACTN3 (rs1815739), ADRB3 (rs4994), CNTFR (rs2070802), and PPARGC1A (rs8192678). Figure 1 showed the results of genotype distribution and allele frequencies of the candidate SNPs in the validation. The genotype distribution was significant in ACTN3 (rs1815739) [Power: RR (37) RX (26) XX (10); Endurance: RR (14) RX (24) XX (14), ${\text{χ}}^{2}$ = 7.812, *p* = 0.020, adj. *p* = 0.05] (Figure 1A), ADRB3 (rs4994) [Power: TT (55) TC (16) CC (2); Endurance: TT (28) TC (20) CC (4), ${\text{χ}}^{2}$ = 6.551, *p* = 0.038, adj. *p* = 0.05] (Figure 1C), and PPARGC1A (rs8192678) [Power: AA (18) AG (38) GG (17); Endurance: AA (6) AG (24) GG (22), ${\text{χ}}^{2}$ = 6.457, *p* = 0.040, adj. *p* = 0.05] (Figure 1G) and not significant in CNTFR (rs2070802) [Power: TT (61) TA (9) AA (3); Endurance: TT (46) TA (2) AA (4), ${\text{χ}}^{2}$ = 3.264, *p* = 0.196, adj. *p* = 0.196] (Figure 1E). The allele frequencies were also significant in ACTN3 (rs1815739) [Power: R (100, 68.5%) X (46, 31.5%); Endurance: R (52, 50.0%) X (52, 50.0%), ${\text{χ}}^{2}$ = 8.715, *p* = 0.003, adj. *p* = 0.012] (Figure 1B), ADRB3 (rs4994) [Power: T (126, 86.3%) C (20,13.7%); Endurance: T (76, 73.1%) C (28,26.9%), ${\text{χ}}^{2}$ = 6.847, *p* = 0.009, adj. *p* = 0.016] (Figure 1D), and PPARGC1A (rs8192678) [Power: A (74, 50.7%) G (72, 49.3%); Endurance: A (36, 34.6%) G (68, 65.4%), ${\text{χ}}^{2}$ = 6.365, *p* = 0.012, adj. *p* = 0.016] (Figure 1H) and not significant in CNTFR (rs2070802) [Power: T (131, 89.7%) A (15, 10.3%); Endurance: T (94, 90.4%) A (10, 9.6%), ${\text{χ}}^{2}$ = 0.029, *p* = 0.864, adj. *p* = 0.864] (Figure 1F).

### Polygenic Models Predict and Identify Power Performance

All variables including candidate SNPs were used for univariate logistic regression in all participants (Table 2). SNPs and phenotypes including gender, age, BMI, SLJ, and SVJ were used for multivariate logistic regression analysis with five models (Table 3). According to the fitted and adjusted effect of the models and the different purposes of talent identification in athletes, model 1 (only three SNPs), model 4 (BMI, SLJ, and three SNPs), and model 5 (BMI, SVJ, and three SNPs) were determined (Table 3). The predictive model contained only three SNPs of ACTN3 (rs1815739), ADRB3 (rs4994), and PPARGC1A (rs8192678), while the identifying model had both three SNPs and phenotypic index.

**TABLE 2:** Univariate logistic regression analysis for power performance.

	All subjects	Power group	Endurance group	OR (95%CI)	p
Participants, *n* (%)	103 (100.0)	60 (58.3)	43 (41.7)	--	--
Gender	--	--	--	--	--
Male, *n* (%)	64 (100.0)	39 (60.9)	25 (39.1)	0.748 (0.334–1.673)	0.479
Female, *n* (%)	39 (100.0)	21 (53.8)	18 (46.2)	--	--
Age, years	24.3 ± 3.2	24.8 ± 3.5	24.7 ± 2.7	1.109 (0.973–1.264)	0.117
Height, cm	179.3 ± 9.3	177.9 ± 8.3	181.2 ± 10.2	0.961 (0.920–1.004)	0.77
Weight, kg	75.8 ± 15.4	76.6 ± 17.4	74.6 ± 12.3	1.009 (0.983–1.035)	0.521
BMI, kg/m^2^	23.4 ± 3.5	24.1 ± 4.3	22.6 ± 1.6	1.159 (1.005–1.336)	0.042
SLJ, cm	289.6 ± 22.2	295.7 ± 22.0	281.0 ± 19.8	1.034 (1.013–1.055)	0.002
SVJ, cm	67.8 ± 14.4	73.5 ± 15.1	59.9 ± 8.3	1.100 (1.052–1.149)	<0.001
ACTN3					
RR, *n* (%)	43 (100.0)	28 (65.1)	15 (34.9)	2.072 (1.150–3.731)	0.015
RX, *n* (%)	45 (100.0)	29 (64.4)	16 (35.6)		
XX, *n* (%)	15 (100.0)	3 (20.0)	12 (80.0)		
ADRB3					
TT, *n* (%)	77 (100.0)	51 (66.2)	26 (33.8)	3.674 (1.475–9.150)	0.005
TC, *n* (%)	25 (100.0)	9 (36.0)	16 (64.0)		
CC, *n* (%)	1 (100.0)	0 (0)	1 (100.0)		
CNTFR (rs2070802)					
TT, *n* (%)	96 (100.0)	55 (57.3)	41 (42.7)	1.084 (0.351–3.352)	0.888
TA, *n* (%)	5 (100.0)	5 (100.0)	0 (0)		
AA, *n* (%)	2 (100.0)	0 (0)	2 (100.0)		
PPARGC1A					
AA, *n* (%)	20 (100.0)	16 (80.0)	4 (20.0)	1.983 (1.085–3.625)	0.026
AG, *n* (%)	53 (100.0)	30 (56.6)	23 (43.4)		
GG, *n* (%)	30 (100.0)	14 (46.7)	16 (53.3)		

**TABLE 3:** Multivariate logistic regression analysis for power performance.

Variants	Model 1		Model 2		Model 3		Model 4		Model 5	
	*β*	p	*β*	p	*β*	p	*β*	p	*β*	p
Gender	--	--	−0.044	0.931	--	--	--	--	--	--
Age	--	--	0.131	0.111	--	--	--	--	--	--
BMI	--	--	0.219	0.020	--	--	0.215	0.018	0.196	0.045
SLJ	--	--	--	--	−0.023	0.228	0.029	0.015	--	--
SVJ	--	--	--	--	0.125	0.001	--	--	0.088	<0.001
ACTN3	0.895	0.007	0.979	0.006	0.785	0.040	0.836	0.021	0.755	0.048
ADRB3	1.266	0.010	1.510	0.006	1.380	0.016	1.451	0.011	1.615	0.011
PPARGC1A	0.770	0.024	0.867	0.019	0.750	0.055	0.836	0.025	0.863	0.034
Constant	−3.651	0.001	−12.481	0.001	−5.289	0.188	−17.176	<0.001	−14.468	<0.001

**FIGURE 2:**
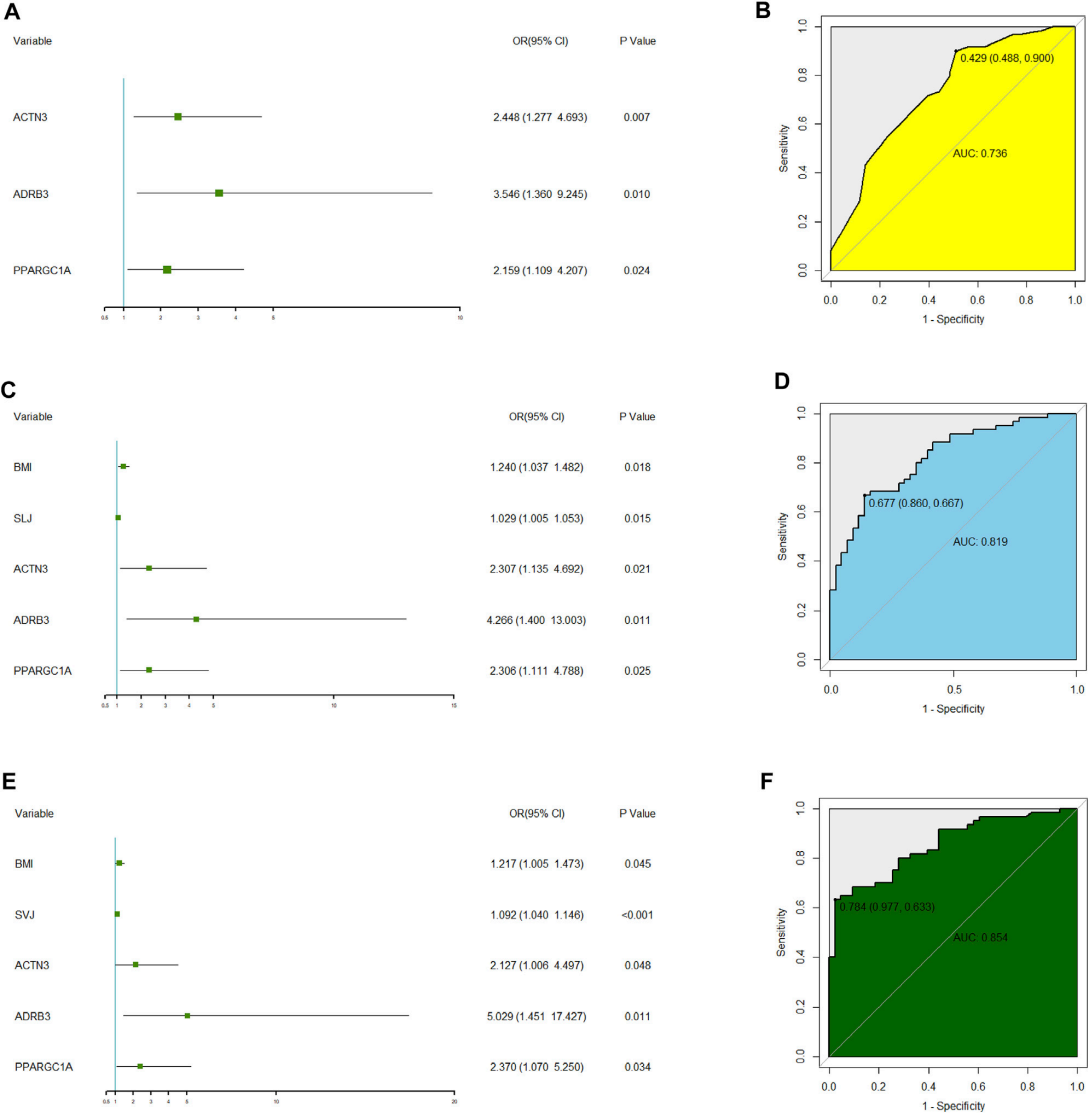
Multivariate logistic regression analysis of three polygenic models related to elite power performance and their ROC analysis. **(A)** Forest plot of multivariate logistic regression analysis for the model1 only including three SNPs. **(B)** Receiver operating characteristic (ROC) curve analysis to summarize the ability of the model1 to predict potential elite power performance from elite endurance performance. AUC: area under the curve. **(C)** Forest plot of multivariate logistic regression analysis for the model 4 including three SNPs, BMI and SLJ. **(D)** Receiver operating characteristic (ROC) curve analysis to summarize the ability of the model4 to identify elite power performance from elite endurance performance. AUC: area under the curve. **(E)** Forest plot of multivariate logistic regression analysis for the model 5 including three SNPs, BMI and SVJ. **(F)** Receiver operating characteristic (ROC) curve analysis to summarize the ability of the model 5 to identify elite power performance from elite endurance performance. AUC: area under the curve.

On multivariate analysis, the predictive model consisted of ACTN3 (rs1815739) (OR = 2.448, 95% CI: 1.277–4.693), ADRB3 (rs4994) (OR = 3.546, 95% CI: 1.360–9.245), and PPARGC1A (rs8192678) (OR = 2.159, 95% CI: 1.109–4.207) (Figure 2A). The predictive model based on the three SNPs fitted the data well (Hosmer–Lemeshow test *p* = 0.873) with a bootstrap-corrected AUC of 0.736 (95% CI: 0.637–0.834) (Figure 2B).

After multiple regression analysis by using SNPs and other phenotypes, two identifying models were obtained (Figure 2C and Figure 2E). The difference between the two models was that one included standing long jump (SLJ) index and the other included standing vertical jump (SVJ) index. Both models had fine goodness of fit (SLJ: Hosmer-Lemeshow test *p* = 0.627; SVJ: Hosmer-Lemeshow test *p* = 0.462). Internally bootstrap-corrected ROC analysis of the two models showed that the AUC value of the model with SVJ (AUC = 0.854, 95% CI: 0.784–0.925, Figure 2F) was higher than that of the model with SLJ (AUC = 0.819, 95% CI: 0.740–0.899, Figure 2D). Therefore, the model including SVJ was selected as the polygenic identifying model of power performance.

**FIGURE 3:**
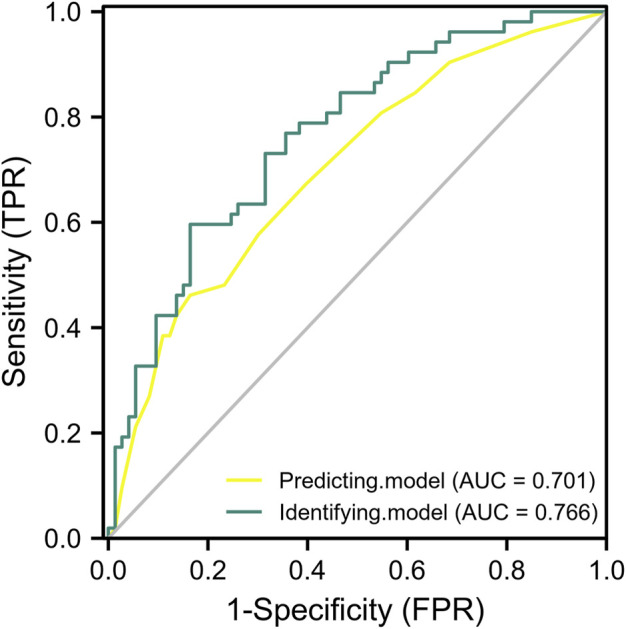
The ROC analysis of predictive and identifying models for cross validation. The yellow curve: the ROC curve of predictive model only including three SNPs. The green curve: the ROC curve of identifying model including three SNPs, BMI, and SVJ.

To verify the external reliability of the models, genotyping data, BMI, and SVJ of another 125 elite athletes were used to crossly validate the predictive and identifying models. After ROC analysis, the AUC of predictive and identifying models were 0.701 (95% CI: 0.609–0.794) and 0.766 (95% CI: 0.683–0.849) (Figure 3). The AUC values of two models were both greater than 0.7, which indicated that the two models had quite good prediction and recognition ability for external data.

### Nomograms of Predictive and Identifying Polygenic Models for Power Performance

**FIGURE 4:**
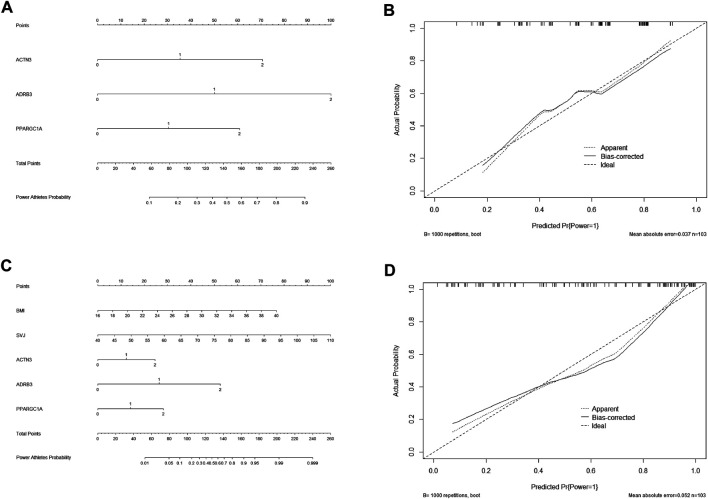
Nomograms of predictive and identifying models for elite power performance and their calibration curves. Genotype value of three SNPs in nomograms: ACTN3, RR = 2 RX = 1 XX = 0; ADRB3, TT = 2 TC = 1 CC = 0; PPARGC1A, AA = 2 AG = 1 GG = 0. **(A)** Nomogram of predictive model (model1 only including three SNPs) for elite power performance. **(B)** Calibration curve of predictive model. **(C)** Nomogram of identifying model (model 5 including three SNPs, BMI and SVJ) for elite power performance. **(D)** Calibration curve of identifying model.

Factors in Figure 2A, including three SNPs of ACTN3 (rs1815739), ADRB3 (rs4994), and PPARGC1A (rs8192678), were used to create an estimation nomogram for predicting power performance. In the receiver operating characteristic (ROC) curve (Figure 2B), the threshold value was 0.429, according to which the cutoff score could be determined in the Nomogram (Figure 4A). Furthermore, a calibration curve graphically showed a good agreement on power performance between nomogram predicting and athletic status (Figure 4B).

The identifying model containing the three SNPs, BMI, and SVJ (Figure 2E), which was also used to develop a nomogram for identifying power performance. The threshold value of ROC curve (Figure 2F) was 0.784, according to which the cutoff score could also be determined in the Nomogram of identifying model (Figure 4C) according to threshold value that was 0.784. The calibration curve also showed a good agreement between nomogram identifying and athletic status (Figure 4D).

### Scores of Two Nomograms

An ROC curve is a plot of the sensitivity versus 1-specificity of a diagnostic test. The different points on the curve correspond to the different cutoff value used to determine whether the results of the test are positive. An ROC curve can be considered the average value of the sensitivity for a test over all possible values of specificity or vice versa (Mandrekar, 2010).

The optimal cutoff value of two nomogram scores were determined to be 130 and 148 by ROC threshold values (0.429 and 0.784) for predicting and identifying power status. The specificity, sensitivity, positive predictive value, negative predictive value, positive likelihood ratio, and negative likelihood ratio were 48.8%, 90.0%, 71.1%, 77.8%, 1.76, and 0.20 in the predictive model, and 97.7%, 63.3%, 97.4%, 65.6%, 27.23, and 0.38 in the identifying model, respectively (Table 4).

**TABLE 4:** Accuracy of two nomograms for predicting and identifying power status.

Variable	Value (95% CI)	
	Predictive model	Identifying model
AUC	0.74 (0.64–0.83)	0.85 (0.78–0.93)
Cutoff score	130	148
Specificity, %	48.8 (33.6–64.3)	97.7 (86.2–99.9)
Sensitivity, %	90.0 (78.8–95.9)	63.3 (49.8–75.1)
Positive predictive value, %	71.1 (59.4–80.6)	97.4 (84.9–99.9)
Negative predictive value, %	77.8 (57.3–90.6)	65.6 (52.6–76.8)
Positive likelihood ratio	1.76 (1.30–2.38)	27.23 (3.89–190.76)
Negative likelihood ratio	0.20 (0.09–0.46)	0.38 (0.27–0.52)

## Discussion

The research on the relationship between gene variants and physical performance started from a single gene and a single SNP. With the expansion of SNP research on candidate genes related to physical performance, an increasing number of SNPs was put into the candidate gene pool. At regular intervals, researchers summarize the progress of such research and new genes (Macarthur and North, 2005; Hagberg et al., 2011; Roth et al., 2012; Perusse et al., 2013; Ahmetov et al., 2016; Sarzynski et al., 2016). However, with the deepening of research, researchers found that single SNP has an insignificant impact on certain physical performance or athletic status. For example, the contribution of ACTN3 R577X polymorphism, which has been studied the most, was estimated to be only 2.5% (Moran et al., 2007). It suggested that it was inadequate to rely on the variant of a single SNP to reflect the impact on a certain performance. The probability of becoming an elite athlete is based on having a set of alleles related to physical performance (Ahmetov et al., 2009; Ruiz et al., 2009). Therefore, it is more appropriate to use the variants of multiple genes or SNPs to reflect the impact on a certain physical performance. Starting from the research of single candidate genes, this study screened several SNPs that may be related to power performance through statistical tests. On this basis, polygenic models were established to reflect the impact on power performance by using multivariate logistic regression. It is more effective to quantify the effect of multiple genes through mathematical methods than that of a single gene alone. Many researchers also use this method to explore the effect of polygenic profiles on physical performance and athletic status (Ruiz et al., 2010; Buxens et al., 2011; Massidda et al., 2014). However, there were some potential problems in these studies, such as the use of the total genotype score (TGS) to reflect the polygenic profiles, but the weight of each SNP in the TGS was equal, without considering the difference of contribution. Additionally, the operability of the predictive/identifying model was relatively poor, which was difficult to be popularized in practice. In our current study, multivariate logistic regression was used to fully consider the contribution weighting of each SNP and nomogram was used to visualize the model and improve the operability, which was more rigorous and feasible than the above-mentioned studies.

ACTN3 R577X gene polymorphism had been repeatedly proven to be associated with elite power performance in many ethnic populations. It is a SNP recognized by most researchers and scholars so far (Tharabenjasin et al., 2019). It was also proven in the East Asian population (Yang et al., 2016; Yang et al., 2017). Compared with ACTN3 R577X, ADRB3 (rs4994) and PPARGC1A (rs8192678) polymorphisms were less reported, especially in the East Asian population.

Beta-Adrenergic receptors are a subgroup of G protein-coupled receptors involved in the regulation of energy metabolism. As a member of the beta-adrenergic receptor family, the ADRB3 (adrenoceptor beta 3) gene locates at 8p11.23 region of the human genome and modulates catecholamine-induced stimulation of adenylate cyclase *via* the action of G proteins (Yang and Tao, 2019). It is generally believed that ADRB3 is mainly expressed in adipocytes and functions to mediate lipolysis and thermogenesis (Collins and Surwit, 2001). The levels of ADRB3 mRNA and protein in the adipose tissue of obese patients and overweight individuals are significantly decreased (Kurylowicz et al., 2015; Cao et al., 2018). Xie et al. (2020) reported that the ADRB3 (rs4994) polymorphism was associated with obesity/overweight during childhood and adolescence in the East Asian population by an evidence-based meta-analysis. They found that children and adolescents with the C allele had an increasing risk of leading to obesity and overweight (Xie et al., 2020). There are few reports on the relationship between ADRB3 (rs4994) polymorphism and elite athletic performance. Santiago et al. (2011) reported that the ADRB3 (rs4994) polymorphism was associated with elite endurance performance in Spanish male athletes. They selected 53 elite power athletes and 100 endurance athletes in Spain as the research objects, and 100 people without sports training experience as the control group. After genotyping the rs4994 locus of ADRB3 gene, they found that the ADRB3 gene was significantly different in the distribution of genotypes among the three groups. Compared with pairs, there was only a significant difference between the endurance group and control group, yet no significance between the power group and endurance group. It was concluded that ADRB3 (rs4994) polymorphism was associated with elite endurance performance (Santiago et al., 2011). Based on the results of statistical analysis, the results of this study were inconsistent with our study, but from the distribution of genotypes in the power group and endurance group, the trend of the two studies was consistent. The inconsistency of the results may be related to ethnic differences (European ancestry populations and East Asian populations) and the sample size.

PPARGC1A (peroxisome proliferator-activated receptor gamma co-activator-1-alpha) is an inducible transcription coactivator, which can participate in many life activities by promoting mitochondrial energy metabolism, such as adaptive thermogenesis, skeletal muscle fiber type conversion, glucose/fatty acid metabolism, and cardiac development (Fontecha-Barriuso et al., 2020). Some studies showed that PPARGC1A gene controls the expression of several genes encoding key enzymes involved in fatty acid oxidation and mainly regulates the induction of muscle adaptation training (Ahmetov et al., 2006). Endurance training can increase PPARGC1A mRNA expression (Franks et al., 2003). In European ancestry populations, carrying A allele was beneficial to the improvement of cardiopulmonary function (Mathai et al., 2008); however, PPARGC1A (rs8192678) polymorphism was not associated with cardiopulmonary function and VO2max in German, Dutch (Stumvoll et al., 2004), and Northern Chinese populations (He et al., 2008), indicating that the relationship between PPARGC1A (rs8192678) gene polymorphism and physical performance was not reproducible in different races and populations, and there was a large ethnic difference. Eynon et al. (2010) studied 155 Israeli athletes and found that the A allele frequency of endurance athletes was low, and GG genotype was conducive to the increase in aerobic capacity. Maciejewska et al. (2012) reported that the frequency of G allele in PPARGC1A gene polymorphism of Polish and Russian athletes was higher than that of the A allele, and concluded that the G allele was the dominant allele and the GG genotype was the dominant genotype for the Polish and Russian endurance athletes. On the surface, the results of the above two studies were opposite to the results of our study on PPARGC1A (rs8192678) polymorphism. The result of this study was that PPARGC1A (rs8192678) polymorphism was associated with power performance, while the other two studies were associated with endurance performance. However, from the detailed data of PPARGC1A (rs8192678) genotype distribution and allele frequency reported in several studies, there was still some consistency. The frequency of the A allele and G allele in the endurance group was 36 and 64%, which was consistent with the results reported by Eynon et al. (2010). If the data of power and endurance groups in our study were combined, the G allele of all athletes would be higher than the A allele, which would be consistent with the results reported by Polish and Russian athletes. Because of the different emphases of research and analysis, the opposite conclusion had been obtained. According to the results of our study, it can also be understood that the G allele of PPARGC1A (rs8192678) polymorphism is associated with endurance performance, while the A allele is associated with power performance.

Most of the candidate SNPs involved in this study were selected from the results reported in English literature, and most of the people were European ancestry populations in Europe and the United States (Ahmetov and Fedotovskaya, 2015; Naureen et al., 2020). The three SNPs in our results have been reported in the Caucasus populations. If the results of this study were directly applied to the Caucasus populations, they should be feasible in theory, but the data of the Caucasus populations should be used for validation. In addition to European ancestry populations, other non-Chinese Han population should be used with caution because there are no relevant research results reported to support it. Due to the large number and complexity of Chinese nationalities and races, it should also be used with caution. The Chinese Han population can use it, while other ethnic minorities should use it with caution before validation.

The purpose of the studies on the multiple genes was applied to predict physical performance development in adolescent athletes and identify sports ability and athletic status in adult athletes, which focused on the relationship between SNP and physical performance and athletic status to create models to apply in talent identification. Only SNP content was involved in the model, so it can only predict the development trend of physical performance in adolescents, but identification power from sports ability and athletic status in adult athletes was insufficient. Our study considered that there was a difference between adolescents and adult athletes in the development potential of physical performance. Adolescent athletes are constantly improving and growing in ability, yet adult athletes, especially elite adult athletes have reached or nearly reached the peak, so the models of two groups should also be different. The model only containing SNPs can be used in adolescent and the other including SNP information and phenotype indicators related to power performance can identify the current situation of sports ability and athletic status in adult athletes. According to the above application objectives, the models in our study were divided into predicting and identifying. BMI and standing vertical jump (SVJ) were added to the identifying model, whose AUC value was improved from 0.736 to 0.854. It showed that the identification power from power performance was improved after phenotypes were added. Compared with previous similar studies (Ruiz et al., 2009; Ruiz et al., 2010; Buxens et al., 2011; Massidda et al., 2014; Ben-Zaken et al., 2015), this study considered the different requirements of the target population for the models, which were more scientific and feasible. The target population includes adolescent and elite athletes. The characteristics of the adolescent population are not obvious before or just upon contact with a sports event, so it is hard to tell which type of sports event is suitable because, at this time, the coaches may need some suggestions about the development trend of power performance to determine the engagement in power-oriented events or not. For the elite athletes engaged in power-oriented events, coaches and scientific research personnel can identify the power status by using the model, so that they can better understand the physical state of athletes and make suitable training and competition plans.

The results of this study can provide insights for research that cannot afford to use sports genomics methods (e.g., GWAS), which is a relatively new scientific discipline focusing on the organization and functioning of the genome of elite athletes (Ahmetov and Fedotovskaya, 2015). Authoritative databases, such as PubMed and Web of Science, can be used to search the genes related to a phenotypic trait and retrieve several SNPs that meet the requirements. If the population to be studied is roughly the same as the population retrieved, SNPs can be directly used as candidates for subsequent research. If the study population is inconsistent with the population retrieved, it can be further screened to eliminate the SNPs that have not been reported or reported no frequency distribution difference, to narrow the range and reduce the cost of research. For example, if a gene is found in the literature, five SNPs of this gene may be associated with phenotypic traits. Further, if it is found that two SNPs have a frequency difference distribution in the study population, and the other three have no frequency difference or no reports, then the two SNPs will be included. From the results of our study, the choice criteria are feasible.

Three SNPs can be tested, genotyped, and given a score on athletes for predicting and identifying power performance. By using two models, the coach can judge athletes’ power performance potential in talent identification and distinguish power performance status in adult athletes to provide a reference for talent identification and sports training. Scores of the three SNPs can be put into predictive model (nomogram) for adolescent athletes. It shows that the athlete has a good development potential of power performance if the total score of the nomogram is more than the 130 cutoff. The measured value of BMI, SVJ, and scores of the three SNPs can be put into the identifying model (nomogram) for adult athletes. It shows that the athlete may have a high level of power performance if the total score of the nomogram is more than the 148 cutoff.

The application of the models will contribute to the fields of talent identification and sports training to a certain extent. Using the cutoff values of 130 and 148, the work of coaches will become intuitive and simple. Coaches can intuitively choose adolescents with a good power performance development potential to engage in power-oriented events when the total score from the nomogram of the predictive model is greater than 130 points. When the total score from the nomogram of the identifying model is greater than 148, it shows that athletes have high level athletic status of power performance, and coaches can reasonably plan sports training programs according to the results.

In conclusion, our results suggest that elite power performance can be predicted and identified by using polygenic profiles in Chinese athletes, according to which, two models have been created and used for talent identification in Chinese athletes. Out of the two models, the predictive one can be used in adolescent athletes to predict the development potential of power performance and the identifying one can be used in elite athletes to distinguish and evaluate power athletic status. They can be applied quickly and visually by using the method of nomogram.

## Data Availability

The original contributions presented in the study are included in the article/Supplementary Materials, further inquiries can be directed to the corresponding authors.
